# Catch-Up Vaccination Intervention and Study of Infant Vaccine Hesitancy in Health District in Palermo (Italy)

**DOI:** 10.3390/vaccines14040366

**Published:** 2026-04-21

**Authors:** Alessandra Fallucca, Roberto Levita, Giuseppe Vella, Angela Sutera, Domenico Mirabile, Antonino Levita, Walter Mazzucco, Francesco Vitale, Alessandra Casuccio

**Affiliations:** 1 Department of Prevention, Provincial Health Authority of Palermo, 90141 Palermo, Italy; alessandra.fallucca@asppalermo.org (A.F.); roberto.levita@asppalermo.org (R.L.); giuseppe.vella@asppalermo.org (G.V.); angela.sutera@asppalermo.org (A.S.); domenico.mirabile@asppalermo.org (D.M.); antonino.levita@asppalermo.org (A.L.); 2 Department of Health Promotion, Mother and Child Care, Internal Medicine and Medical Specialties, University of Palermo, 90127 Palermo, Italy; walter.mazzucco@unipa.it (W.M.); francesco.vitale@unipa.it (F.V.)

**Keywords:** vaccination coverage, vaccine hesitancy, catch-up vaccination intervention, health action process approach, health literacy

## Abstract

Background: Despite the introduction in 2017 of mandatory vaccination for the hexavalent and the measles–mumps–rubella–varicella vaccines, childhood vaccination coverage in Sicily (Italy) remains below the recommended and safety threshold of 95%. A catch-up vaccination intervention was implemented for the pediatric population of the 2022–2023 birth cohorts residing in a health district of Palermo (Bagheria) where in 2024, 24-month coverage for polio and measles was 77.29% and 77.62%, respectively. Methods: A cross-sectional study with a before–after component was conducted between June 2025 and December 2025, with the aim of evaluating the increase in vaccination coverage. A questionnaire was administered to the parents of non-compliant children to investigate the determinants of infant vaccine hesitancy. Results: Collaboration with primary care pediatricians and the organization of active call sessions and extra vaccination sessions resulted in an increase in vaccination coverage of approximately 10–12 percentage points in both birth cohorts. The investigation of the determinants of vaccination adherence showed some significant associations: “perception of infectious disease risk” (OR: 7.91; *p* = 0.009) and “expectations of a positive outcome from vaccination” (OR: 8.62; *p* = 0.003). Vaccine information sources such as the internet and media were associated with refusal of catch-up vaccination (OR: 0.47, *p* < 0.001; and OR: 0.13, *p* = 0.026, respectively). Conclusions: Despite methodological limitations, such as the self-reported nature of the survey data, the study demonstrated the usefulness of local strategies aimed at vaccination catch-up, representing a valuable example of local public health practice and effectively contributing to improved vaccination coverage in the pediatric population.

## 1. Introduction

Vaccination programs are an essential public health tool designed to ensure high immunization coverage in the population, providing both individual and collective protection. The effectiveness of immunization programs is based on a high level of adherence by the population and requires a minimum threshold of 95%, as recommended by the World Health Organization [1,2]. Over the past decade, maintaining adequate vaccination coverage in Italy has represented a crucial public health challenge, as several pediatric immunization rates have declined below the safety threshold [3]. This reduction has contributed to the progressive re-emergence of vaccine-preventable diseases. Within this framework, several waves of marked increases in measles incidence have been observed in recent years starting in 2017, consistent with a nationwide epidemic (over 5000 reported cases across the country), with the most recent outbreaks of 1055 and 532 cases reported in 2024 and 2025 respectively [4,5].

In response to declining vaccination coverage, Italy’s Decree-Law No. 119 of 2017 introduced mandatory vaccination for children aged 0 to 16. In Italy, the 2017 mandatory vaccination law also introduced specific consequences for non-compliance. In particular, vaccination status is required for access to nurseries and kindergartens, and financial penalties may be applied to families who do not comply with the vaccination schedule.

Mandatory vaccinations include the hexavalent vaccine, which protects against polio, diphtheria, tetanus, hepatitis B, pertussis, and *Haemophilus influenzae* type B, with a three-dose schedule within the first year of life and subsequent boosters, and the MMRV vaccine, which protects against measles, mumps, rubella, and varicella, with a first dose to be administered by age two and a subsequent booster according to the recommended schedule [6].

Following the implementation of mandatory vaccination, coverage rates for polio (a proxy for the hexavalent vaccine) and measles (a proxy for the MMRV vaccine) have fluctuated around 95%. In 2018, the national average coverage at 24 months was 95.15% for polio and 94.49% for measles [3]. Despite mandatory vaccination, vaccination coverage remains below the recommended threshold in some regions, highlighting the persistence of structural and behavioral barriers. Significant regional disparities can be observed, with rates in the southern regions falling below the national average. In Sicily, measles vaccination coverage at 24 months was 90.91% in 2018, showing a declining trend in recent years; in 2023, 24-month measles coverage was 90.79% (2021 cohort), while in 2024 it further decreased to 87.98% (2022 cohort) [3,7]. The geographic variation and declining trend of coverage observed in Sicily underscore the need for targeted interventions to address gaps in vaccination coverage and counter vaccine hesitancy.

Vaccine catch-up is an essential strategy for identifying and vaccinating individuals who have not completed their vaccination schedule within the recommended timeframe. According to the WHO, a well-defined and planned catch-up policy is an integral part of an effective national immunization program [8]. There are many different approaches to vaccination catch-up campaigns, and they are often implemented in combination through multi-component or multimodal interventions in order to maximize vaccination uptake. Among the most effective strategies, highlighted in the scientific literature, are: “reminders”, sending letters or emails to the unvaccinated population; “active calls”, to propose missed vaccinations; “extraordinary vaccination sessions,” with extended hours or special opening days; and “incentives” to stimulate vaccination demand [9].

Italy’s National Immunization Program (PNPV 2023–2025) promotes catch-up efforts to reduce inequalities, improve vaccination coverage through targeted interventions for at-risk groups, and address vaccine hesitancy. Vaccine hesitancy, defined as the delay or refusal of vaccines despite their availability, has been listed by WHO as a major global health threat, hindering vaccination efforts and reversing progress against preventable diseases [10]. It is a complex and dynamic issue influenced by individual, socio-demographic, and behavioral factors [11,12]. The literature also identifies a lack of awareness of the benefits of vaccination, fear of adverse effects, and inadequate health literacy as key factors contributing to vaccine hesitancy. Several studies investigating vaccination uptake have employed validated cognitive–behavioral constructs and health literacy measures [13,14,15].

This study was conducted with the main aim of evaluating the effectiveness of a structured catch-up vaccination program targeting the 2022 and 2023 birth cohorts in a health district of Palermo (DS-39 PA; Bagheria), where the vaccine coverage for infant immunization schedule was approximately 10% less than the regional one. The study also investigated the determinants of infant vaccine hesitancy, exploring cognitive–behavioral models and health literacy.

## 2. Materials and Methods

### 2.1. Study Design

The study included two complementary components. First, a cross-sectional design was used to investigate vaccine hesitancy and its determinants among parents of non-compliant children residing in a health district of Palermo. Second, a before–after (quasi-experimental) approach was applied to evaluate changes in vaccination coverage at the population level following the implementation of the multimodal catch-up intervention.

The study was conducted from June to December 2025 at the Bagheria Vaccination Center. The Bagheria Health District (DS-39 PA) is one of the most densely populated peripheral areas in the Palermo territory, with a total of approximately 100,000 residents and an average of 900 births per year across the district [16].

### 2.2. Study Population

The study assessed the population of the 2022–2023 birth cohorts with the registration status of “Resident” or “New Resident/Immigrant” in the Bagheria health district. Overall, the pediatric population with these demographic characteristics amounted to 1748 subjects (916 children from the 2022 birth cohort and 832 from the 2023 cohort). Vaccination coverage data for the Bagheria health district were extracted from the Unified Regional Vaccination Registry (AVUR). Polio vaccination coverage before the catch-up intervention was 77.29% for the 2022 cohort and 78.18% for the 2023 cohort. Measles vaccination coverage before the catch-up intervention was 77.62% for the 2022 cohort and 78.32% for the 2023 cohort. The population not compliant with vaccination was identified by cross-referencing data extracted from AVUR with paper documentation (clinical reports and individual vaccination booklets).

### 2.3. Vaccinal Catch-Up Intervention

A multimodal catch-up intervention was implemented by the Department of Prevention of the Provincial Health Authority of Palermo through collaboration with primary care pediatricians, who played a key role in facilitating the retrieval of telephone numbers and email addresses of families of non-compliant children. Active calls were conducted to contact the families, and, following those, reminder emails were sent to confirm the scheduled appointments. For each family, at least two attempts were made to contact the family by telephone and to make an active call. Families were invited to attend the vaccination center in Bagheria during dedicated extra vaccination sessions, organized to expand service availability and improve vaccination uptake. Overall, five additional vaccination sessions were carried out. At the end of the catch-up intervention, vaccination coverage data for the 2022–2023 birth cohorts residing in the Bagheria health district were extracted and assessed.

### 2.4. The Questionnaire

During vaccination sessions, a questionnaire developed in collaboration with the School of Specialization in Public Health and Preventive Medicine at the University of Palermo was administered to identify the factors that determine vaccine hesitation. Before completing the questionnaire, parents were informed of its anonymous and voluntary nature, and they provided informed consent by signing their signature, according to privacy regulation (GDPR 2016/679). The questionnaire investigated several factors: socio-demographic aspects (educational level, economic status, and family members), lifestyle habits (smoking and healthy diet), health status and comorbidities, and sources of information about vaccination. Socio-demographic variables were classified based on the data declared by the interviewees, such as education level and economic status, while smoking and fruit and vegetable consumption were assessed according to national and international guidelines [17,18].

To investigate the cognitive–behavioral determinants associated with adherence to catch-up vaccination, a previously validated Health Action Process Approach (HAPA) construct was used [15,19]. The HAPA model was applied using eight items to investigate four distinct areas: the “perception of risk” of vaccine-preventable diseases, the “expectations of positive” and “negative” outcomes of vaccination, and “self-efficacy”. Responses, on a five-point Likert scale, ranged from 1 = “I completely disagree” to 5 = “I completely agree.” For each respondent, the scores for the two items within the same domain were summed (with a score ranging from 2 to 10 per domain), and the median value was calculated. Scores greater than or equal to the median were associated with a “high” level of awareness for each HAPA domain; lower scores were associated with a “low” level.

The questionnaire also included two tests used to assess health literacy: the SILS (Single Item Literacy Screener) Test and the METER Test (Medical Term Recognition Test) [20,21,22]. The first one consisted of a question exploring the need for assistance in reading medical materials, with a score ranging from “never” (high level) to “always” (low level). The second test assessed the ability to recognize medical terms, requiring the correct identification of terms from a list of 70 words. The results were classified into three levels of health literacy: low (0–20 correct terms), marginal (21–34), and adequate (35–40). The study was approved by the Local Ethics Committee of Palermo (CEL-1) on 8 July 2025.

### 2.5. Statistical Analysis

Statistical analysis was conducted using Stata/SE 14.2 software. The normality of quantitative variables was assessed using skewness and kurtosis tests. Variables with a normal distribution were described using the mean and standard deviation (SD), while those with a non-normal distribution were reported as the median and interquartile range (IQR). Qualitative variables were summarized using absolute and relative frequencies.

Comparisons between groups for quantitative variables were performed using Student’s *t*-test for normally distributed variables and the Wilcoxon test for non-normally distributed variables; for qualitative variables, the Chi-square test was used.

Vaccination coverage data before and after the intervention were analyzed descriptively at the population level. As individual-level paired data were not available for the entire study population, statistical tests for paired comparisons (e.g., the McNemar test) were not applicable.

Additionally, univariate logistic regression analysis was performed to identify factors associated with acceptance of the proposed vaccination. A multivariate logistic regression model was constructed including variables with a *p*-value ≤ 0.05 in the univariate analysis. A *p*-value ≤ 0.05 was considered statistically significant for all analyses.

## 3. Results

### 3.1. Descriptive Analysis: Intercepted Pediatric Population

**Figure 1:**
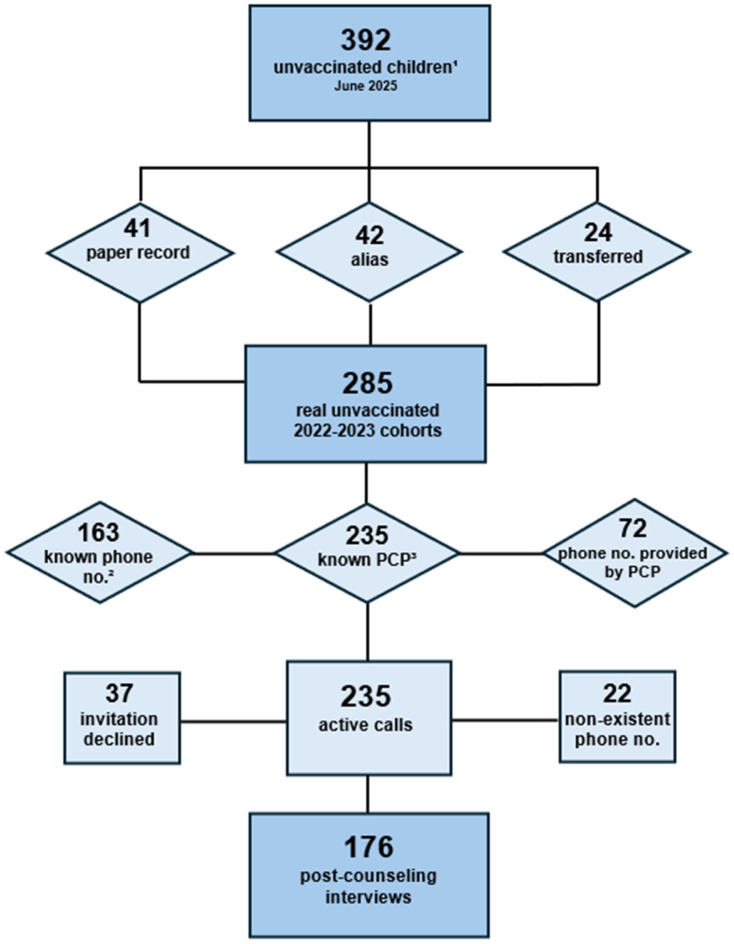
Flow-chart of the intercepted pediatric population non-compliant with vaccinations. Unvaccinated children^1^: population of the 2022–2023 birth cohorts not vaccinated with three doses of hexavalent vaccine or with the MMRV vaccine; phone no.^2^: telephone number of the unvaccinated population; PCP^3^: primary care pediatrician.

Analysis of data extracted from AVUR revealed 22.14% of the study population were not protected with three doses of the hexavalent vaccination (*n* = 387; 208 children from the 2022 cohort and 179 children from the 2023 cohort), and 21.57% were not protected with the first dose of the anti-MMRV vaccination (*n* = 377; 208 children from the 2022 cohort and 172 from the 2023 cohort). Many children were non-compliant with both vaccinations, and by cross-referencing the data from the two databases, a total of 392 non-compliant children were identified and required intervention. A cross-check of the paper vaccination records revealed that vaccination records were missing from the electronic archive for 41 children who were up to date. A verification of personal data identified 42 “alias” identities—that is, duplicate identifiers for the same individual, characterized by alternative personal details—and 24 children who had moved out of the region and were no longer residents of the Bagheria health district. As a result of these findings, a total of 107 children were excluded from the study (Figure 1).

The resulting target population consisted of 285 children and was further investigated. For each child non-compliant with vaccinations, the availability of phone numbers and email addresses was verified. For 72 children, the phone number was provided by the primary care pediatrician. A total of 253 active calls were made to discuss catch-up vaccination; in 59 cases, it was not possible to reach the families (37 invitations declined and 22 non-existent/unreachable telephone numbers) (Figure 1).

Overall, 176 families accepted the invitation to visit the vaccination center, representing the final sample of post-vaccination counseling interviews (Figure 1).

### 3.2. Descriptive Analysis: Catch-Up Vaccination Coverage

The families of 176 children who were not compliant with vaccinations were invited to the Bagheria vaccination center for counseling and active vaccination promotion. Overall, 72.16% of parents (*n* = 127) accepted catch-up vaccination for their child; in 27.16% of cases, vaccination was refused and a written denial was required (*n* = 49).

**Figure 2:**
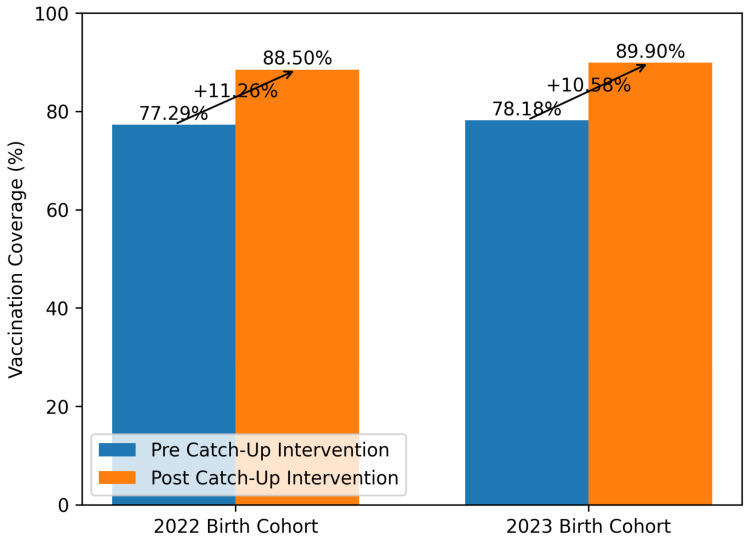
Catch-up polio vaccination coverage for the 2022–2023 birth cohorts. Data are expressed as a percentage (%). The *y*-axis ranges from 0 to 100%. The polio vaccine was used as a proxy for the hexavalent vaccine—polio, diphtheria, tetanus, hepatitis B, pertussis, and Haemophilus influenzae type B; vaccination coverage data were extracted from the Regional Single Vaccination Registry in June 2025 (pre-intervention) and in December 2025 (post-intervention).

Vaccination coverage data extracted before the catch-up intervention showed polio coverage of 77.29% for the 2022 birth cohort and 78.18% for the 2023 birth cohort. Post-intervention, vaccination coverage had increased to 88.50% and 89.90% for the 2022 and 2023 cohorts, respectively. The analysis of vaccination coverage data for polio before and after the catch-up campaign revealed an increase of 11.26% for the 2022 birth cohort and 10.58% for the 2023 birth cohort (Figure 2).

Vaccination coverage data extracted before the catch-up intervention showed measles coverage of 77.62% for the 2022 birth cohort and 78.32% for the 2023 birth cohort.

**Figure 3:**
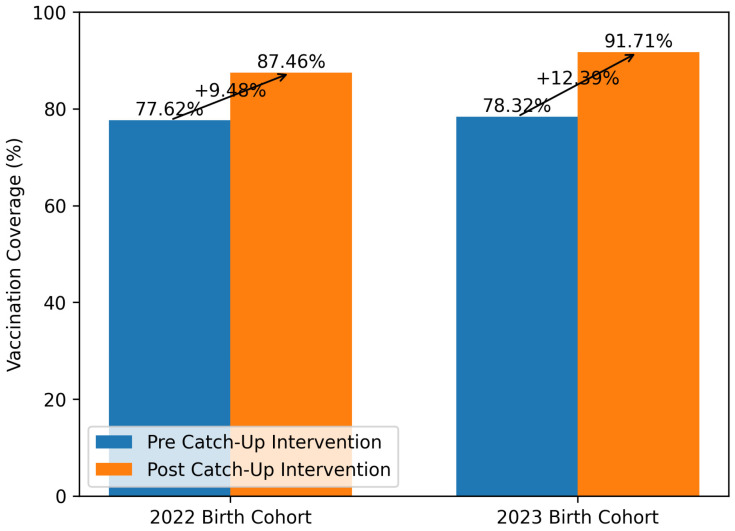
Catch-up measles vaccination coverage for the 2022–2023 birth cohorts. Data are expressed as a percentage (%). The *y*-axis ranges from 0 to 100%. The measles vaccine was used as a proxy for the MMRV (measles, mumps, rubella, and varicella) vaccine; vaccination coverage data were extracted from the Regional Vaccination Registry in June 2025 (pre-) and in December 2025 (post-intervention).

The measles vaccination coverage rate observed after the intervention was 87.46% for the 2022 birth cohort and 91.71% for the 2023 birth cohort. Overall, the catch-up intervention resulted in an increase in measles vaccine coverage of 9.48% for the 2022 birth cohort and 12.39% for the 2023 birth cohort (Figure 3).

### 3.3. Descriptive Analysis: Characteristics of the Population Non-Compliant with Vaccinations

The parents of 176 out of 392 children involved in the catch-up intervention intercepted by the active call were administered a questionnaire. The survey involved both parents who accepted catch-up vaccination for their child and those who, despite recommendations, refused the vaccine by signing the required legal denial form. Most parents indicated the primary care pediatrician as their primary source of information on pediatric vaccinations (*n* = 110; 62.50%). The following sources were most frequently cited as sources of information on vaccines: the internet (*n* = 53; 30.11%), family members and relatives (*n* = 41; 23.30%), medical specialists (*n* = 31; 17.61%), and health education brochures provided by the local public health service (*n* = 30; 17.05%) (Table 1).

The main reason for most of the interviewees for not vaccinating their child according to the schedule set by the vaccination schedule was fear of side effects (*n* = 70; 42.71%), followed by distrust of vaccinations (*n* = 40; 22.73%). The majority of parents reported no pathological conditions for their child (*n* = 110; 62.50%); 7.95% (*n* = 14) were affected by respiratory pathologies (i.e., frequent rhinitis, asthma, and bronchitis), 7.39% (*n* = 13) by disabilities (i.e., cognitive and/or motor developmental delay), and 6.25% (*n* = 11) by autism (Table 1).

The investigation on socio-demographic characteristics showed that approximately two-thirds of the interviewees had obtained an educational qualification higher than middle school (*n* = 106; 60.23% had a high school diploma or university degree). In most cases, the household consisted of four or more members (*n* = 114; 64.77%). A significant portion of the interviewed parents declared to be smokers (*n* = 70; 39.77%); 31.25% reported having never smoked (*n* = 55). Most respondents reported consuming three or four portions of fruits and vegetables per day (*n* = 79; 44.89%), followed by one or two portions per day (*n* = 68; 38.64%) (Table 2).

Analysis of cognitive–behavioral factors (HAPA model) showed that approximately half of the parents interviewed had an accurate perception of the risk associated with vaccine-preventable infectious diseases (*n* = 96; 54.55%); most demonstrated a high degree of expectation of a positive outcome from vaccination (*n* = 118; 67.05%) and of self-efficacy, defined as the ability to carry out a preventive action one intends to perform (*n* = 132; 75.00%) (Table 3).

The assessment of the level of health literacy showed that although approximately one-third of the respondents had answered in the SILS Test that they “never” (*n* = 14; 7.95%) or “rarely” (*n* = 42; 29.55%) needed help in understanding medical texts or instructions, in reality, the METER Test found that the majority of the respondents had a “marginal” (*n* = 84; 47.73%) or “low” (*n* = 71; 40.34%) level of health literacy (Table 3).

Stratified frequency analysis revealed characteristic differences between parents who consented to catch-up vaccination and those who refused. Specifically, the primary care pediatrician was a more frequent source of information for vaccinated children compared to unvaccinated children (70.08% vs. 42.86%; *p* = 0.001). Health education brochures provided by the local public health service were also a more frequent source of information among families of vaccinated children compared to unvaccinated children (21.26% vs. 6.12%; *p* = 0.0017). On the other hand, the internet and media were more frequent sources among families who refused catch-up vaccination: 16.54% vs. 65.31%, respectively, with *p* < 0.001; and 7.87% vs. 30.61%, respectively, with *p* < 0.001. Distrust in vaccination was the most frequently cited reason by parents who refused catch-up vaccination (18.11% vs. 34.69%; *p* = 0.030); children who were vaccinated after the catch-up activity were more frequently found to be healthy and not affected by any pathology (73.23% vs. 34.69%; *p* < 0.001). Catch-up vaccination was more frequently refused by parents of children with disabilities and autism (1.57% vs. 22.45%, *p* < 0.001; and 0.79% vs. 20.42%, *p* < 0.001, respectively) (Table 1).

The comparison between vaccinated and unvaccinated children showed that parents with low educational levels were more likely to refuse catch-up vaccination (31.50% vs. 61.22%; *p* < 0.001). In contrast, higher daily fruit and vegetable consumption was associated with a higher rate of adherence to catch-up vaccination (18.90% vs. 4.08%; *p* = 0.027) (Table 2).

Furthermore, the stratified analysis showed that those who adhered more to catch-up vaccination for their children were those parents with a higher degree of perception of the risk related to infectious diseases preventable by vaccination (66.14% vs. 24.49%, *p* < 0.001), with a higher level of expectations of a positive outcome from vaccinations (75.59% vs. 44.90%; *p* < 0.001) and with adequate awareness of post-vaccination adverse events and self-efficacy (80.31 vs. 38.78, *p* < 0.001, and 80.31% vs. 61.22%, *p* = 0.009, respectively). Parents who achieved an adequate level of health literacy on the METER Test more frequently agreed to the offer of catch-up vaccination (14.96% vs. 4.08; *p* < 0.001); on the other hand, parents with a low METER score more frequently refused vaccination (29.13% vs. 69.29%; *p* < 0.001), as did parents who answered “often” or “always” on the SILS Test (16.54% vs. 42.86%, *p* = 0.006, and 6.30% vs. 14.29%, *p* < 0.001) (Table 3).

### 3.4. Logistic Analysis of the Determinants of Adherence to Catch-Up Vaccination

In multivariate analysis, the determinants of pediatric catch-up vaccination program adherence were found to be two domains of the HAPA cognitive–behavioral model: a high level of perceived risk related to vaccine-preventable infectious diseases (OR: 7.91; *p* = 0.009) and a high degree of positive expectations regarding the protective effect of vaccinations (OR: 8.62; *p* = 0.003). On the other hand, using the internet (OR: 0.47; *p* < 0.001) or media (OR: 0.13; *p* = 0.026) as the primary source of information about vaccinations and the presence of cognitive or motor disabilities in the child (OR: 0.01; *p* = 0.011) were negatively associated with catch-up vaccination adherence. Smoking habits were also negatively associated with vaccination adherence, especially among parents who reported having recently quit (OR: 0.09; *p* = 0.002) (Table 4).

## 4. Discussion

Catch-up vaccination activities are essential strategies to restore vaccination coverage in populations that have not completed the recommended schedule. These interventions, recommended by the WHO and included in the Italian National Immunization Plan (PNPV), are particularly effective in settings where childhood vaccination coverage is suboptimal [1,8]. In this context, our study aimed to implement a structured catch-up intervention targeting non-compliant children from the 2022–2023 birth cohorts in a health district of Palermo.

Our findings are consistent with previous evidence demonstrating the effectiveness of multimodal strategies—including active calls, reminders, and extended vaccination sessions—in improving vaccination coverage [9]. Systematic reviews have shown that tailored, community-based interventions can significantly increase vaccine uptake, particularly in populations with low baseline coverage [23,24].

In line with the literature, our results confirm the central role of behavioral determinants in vaccination decisions. Risk perception and expectations of positive outcomes were significantly associated with vaccine acceptance, as described in studies applying cognitive–behavioral models such as the Health Action Process Approach (HAPA) [15,19]. Additionally, reliance on non-institutional information sources, such as the internet and media, was negatively associated with vaccination adherence, supporting previous evidence linking these channels to increased vaccine hesitancy [25,26].

Compared with previous studies, our research provides an integrated perspective by combining real-world intervention outcomes with behavioral and health literacy factors, offering practical insights for the design of targeted public health strategies.

The intervention resulted in a substantial increase in vaccination coverage for both polio and measles, with gains of approximately 10–12 percentage points over a 7-month period. These findings are consistent with previous studies reporting significant improvements in vaccination uptake following catch-up interventions [9,27]. Although the 95% coverage threshold was not achieved, the observed increase supports the effectiveness of proactive, community-based approaches based on active recall and interprofessional collaboration. These results have encouraged the extension of the intervention to the broader region and to subsequent birth cohorts.

A key element of the intervention was the collaboration with primary care pediatricians, which proved essential in identifying and contacting non-compliant families. Pediatricians represent a trusted source of information and play a strategic role in promoting vaccination through personalized counseling and follow-up of vaccination status. Their influence on parental decision-making has been widely documented in the literature [28,29].

The analysis of information sources confirmed that the internet was frequently associated with lower adherence to vaccination, highlighting the impact of misinformation. Fear of adverse effects and distrust in vaccines emerged as the main reasons for refusal, emphasizing the importance of effective communication strategies. In this context, healthcare professionals, particularly pediatricians, play a crucial role in counteracting misinformation and supporting informed decision-making [14,30,31,32].

Another factor negatively associated with vaccination adherence was the presence of disability. Although individuals with comorbidities are at increased risk of vaccine-preventable diseases, barriers such as accessibility issues, lack of tailored communication, and parental concerns may reduce vaccination uptake. These findings are consistent with previous studies highlighting lower vaccination coverage among children with special needs [33,34,35,36]. Targeted strategies, including personalized counseling and accessible vaccination services, are therefore needed to address these gaps.

The analysis of cognitive–behavioral determinants confirmed the relevance of two key HAPA domains: risk perception and positive outcome expectations. Parents who perceived a higher risk of infectious diseases and recognized the benefits of vaccination were more likely to accept catch-up vaccination. These findings reinforce the role of cognitive factors in shaping vaccination behavior and suggest that integrating messages on the benefits of vaccination into pediatric campaigns may improve adherence, even in hesitant populations [15,37,38,39].

Although the relationship between catch-up interventions and behavioral determinants has been previously described, our findings contribute real-world evidence from a low-coverage setting. The integration of intervention outcomes with behavioral and health literacy factors allows for a more comprehensive understanding of vaccine uptake dynamics.

From a public health perspective, the sustainability of the intervention is a key aspect. The multimodal strategy implemented in this study could be extended to subsequent birth cohorts through integration into routine vaccination services. In particular, collaboration with primary care pediatricians, active call systems, and dedicated vaccination sessions could be institutionalized within immunization programs.

Such an approach would enable periodic catch-up interventions targeting newly eligible cohorts or populations with delayed vaccination schedules, contributing to the maintenance of adequate vaccination coverage over time. Future studies should evaluate the long-term effectiveness and cost-effectiveness of these strategies on a larger scale.

Vaccination coverage is also influenced by national immunization policies. In several countries, policies linking immunization to school enrollment have contributed to maintaining high coverage levels. In Italy, the 2017 mandatory vaccination law strengthened access requirements; however, regional disparities persist, particularly in southern areas such as Sicily [6].

This suggests that mandatory policies alone may not be sufficient to ensure optimal vaccination coverage and that complementary strategies addressing behavioral and access-related barriers are needed. In this context, our findings support the role of targeted, community-based interventions as effective tools to improve vaccination uptake in low-coverage settings.

Despite these relevant findings, some limitations should be considered when interpreting the results. Data were collected through questionnaires administered to parents, and self-reported responses may be subject to recall bias. The study population was identified using the Regional Vaccination Registry (AVUR), which may not always be promptly updated, particularly in cases of changes in residence or registry inaccuracies (e.g., duplicate or “alias” records); therefore, vaccination coverage data may be biased by incomplete or delayed updates.

Furthermore, the intervention targeted the non-compliant pediatric population of two birth cohorts within a single health district; therefore, the study is limited by a relatively small and geographically restricted sample.

An additional limitation of this study concerns the proportion of families who could not be reached during the active call phase. Approximately 20% of the target population was not contactable, which may have introduced a selection bias. It is possible that these families differ systematically from those who were reached in terms of socio-demographic characteristics, access to healthcare services, or attitudes toward vaccination. As a result, the observed effects of the intervention may not be fully generalizable to the entire target population. However, the use of population-level vaccination coverage data allows for a more comprehensive assessment of overall trends.

Despite these limitations, the present study not only assessed the effectiveness of a catch-up vaccination intervention but also provided a comprehensive analysis of its real-world implementation, serving as a replicable pilot experience. Furthermore, some novel aspect of the study were: (i) the evaluation of a structured, multimodal catch-up intervention in a low-coverage setting; (ii) the integration of intervention outcomes with behavioral determinants within the framework of the Health Action Process Approach (HAPA); (iii) the identification of context-specific factors, including information sources, that influenced vaccine acceptance.

## 5. Conclusions

The study demonstrated the effectiveness of a structured catch-up vaccination program for a pediatric population, aimed at improving vaccination coverage. The multimodal intervention, involving active call-out activities, personalized counseling, special vaccination sessions, and collaboration with primary care pediatricians, demonstrated that targeted and organized interventions may significantly increase vaccination coverage in the short term. The project also identified several determinants of catch-up vaccination compliance, including a high perception of infectious disease risk, adequate expectation of a positive outcome, and the role of vaccination information sources.

## Figures and Tables

**Table 1:** Characteristics of the population: sources of information, pathologies and reasons for non-vaccination.

General Characteristics		Total Respondents *n* (%)		Vaccinated *n* (%)		Unvaccinated *n* (%)		*p*-Value *
		176 (%)		127 (72.16)		49 (27.84)		
**Source of information about vaccines**								
Primary care pediatrician	No	66	(37.50)	38	(29.92)	28	(57.14)	0.001
	Yes	110	(62.50)	89	(70.08)	21	(42.86)	
Family	No	135	(76.70)	101	(79.53)	34	(69.39)	0.154
	Yes	41	(23.30)	26	(20.47)	15	(30.61)	
Media	No	151	(85.80)	117	(92.13)	34	(69.39)	<0.001
	Yes	25	(14.20)	10	(7.87)	15	(30.61)	
Friends/Colleagues	No	168	(95.45)	101	(95.28)	47	(95.92)	0.854
	Yes	8	(4.55)	26	(4.72)	2	(4.08)	
Specialist physicians	No	145	(82.39)	121	(79.53)	44	(89.80)	0.109
	Yes	31	(17.61)	6	(20.47)	5	(10.20)	
Health education brochures	No	146	(82.95)	100	(78.74)	46	(93.88)	0.017
	Yes	30	(17.05)	27	(21.26)	3	(6.12)	
Internet	No	123	(69.89)	106	(83.46)	17	(34.69)	<0.001
	Yes	53	(30.11)	21	(16.54)	32	(65.31)	
**Reason for non-vaccination**								
Lack of information		18	(10.23)	12	(9.45)	6	(12.24)	0.030
Access barriers		25	(14.20)	21	(16.54)	4	(8.16)	
Lack of awareness		18	(10.23)	17	(13.39)	1	(2.04)	
Fear of adverse effects		75	(42.61)	54	(42.52)	21	(42.86)	
Distrust in vaccines		40	(22.73)	23	(18.11)	17	(34.69)	
**Pathologies of the child**								
Respiratory conditions	No	162	(92.05)	115	(90.55)	47	(95.92)	0.238
	Yes	14	(7.95)	12	(9.45)	2	(4.08)	
Immunological conditions	No	171	(97.16)	124	(97.64)	47	(95.92)	0.538
	Yes	5	(2.84)	3	(2.36)	2	(4.08)	
Autoimmune diseases	No	168	(95.45)	121	(95.28)	47	(95.92)	0.854
	Yes	8	(4.55)	6	(4.72)	2	(4.08)	
Genetic conditions	No	167	(94.89)	122	(96.06)	45	(91.84)	0.254
	Yes	9	(5.11)	5	(3.94)	4	(8.16)	
Autism	No	165	(93.75)	126	(99.21)	39	(79.59)	<0.001
	Yes	11	(6.25)	1	(0.79)	10	(20.41)	
Disabilities	No	163	(92.61)	125	(98.43)	38	(77.55)	<0.001
	Yes	13	(7.39)	2	(1.57)	11	(22.45)	
No pathology	No	66	(37.50)	34	(26.77)	32	(65.31)	<0.001
	Yes	110	(62.50)	93	(73.23)	17	(34.69)	

* For categorical variables with multiple categories, *p*-values refer to the overall distribution across categories.

**Table 2:** Socio-demographic characteristics of the population and lifestyle.

Socio-Demographic Characteristics and Lifestyle	Total Respondents *n* (%)		Vaccinated *n* (%)		Unvaccinated *n* (%)		*p*-Value *
	176		127 (72.16)		49 (27.84)		
**Family members**							
≤4	114	(64.77)	84	(66.15)	30	(61.23)	0.648
>4	62	(35.23)	43	(33.85)	19	(38.77)	
**Educational level**							
Low level	70	(39.77)	40	(31.50)	30	(61.22)	<0.001
High level	106	(60.23)	87	(68.50)	19	(38.78)	
**Smoker**							
No, never smoker	55	(31.25)	46	(36.22)	9	(18.37)	0.110
No, quit smoking more than 10 years ago	20	(11.36)	15	(11.81)	5	(10.20)	
No, quit smoking less than 10 years ago	31	(17.62)	20	(15.75)	11	(22.45)	
Yes, currently smoking	70	(39.77)	46	(36.22)	24	(48.98)	
**Daily fruit and vegetable intake**							
No portions	3	(1.70)	2	(1.57)	1	(2.04)	0.027
1 or 2 portions per day	68	(38.64)	42	(33.07)	26	(53.06)	
3 or 4 portions per day	79	(44.89)	59	(46.46)	20	(40.82)	
≥5 portions	26	(14.77)	24	(18.90)	2	(4.08)	

* For categorical variables with multiple categories, *p*-values refer to the overall distribution across categories.

**Table 3:** Health Action Process Approach (HAPA) and health literacy.

		Total Respondents *n* (%)		Vaccinated *n* (%)		Unvaccinated *n* (%)		*p*-Value *
		176		127 (72.16)		49 (27.84)		
**Risk Perception**								
	Low level	80	(45.45)	43	(33.86)	37	(75.51)	<0.001
	High level	96	(54.55)	84	(66.14)	12	(24.49)	
**Positive Outcome**								
	Low level	58	(32.95)	31	(24.41)	27	(55.10)	<0.001
	High level	118	(67.05)	96	(75.59)	22	(44.90)	
**Negative Outcome**								
	Low level	55	(31.25)	25	(19.69)	30	(61.22)	<0.001
	High level	121	(68.75)	102	(80.31)	19	(38.78)	
**Self-efficacy**								
	Low level	44	(25.00)	25	(19.69)	19	(38.78)	0.009
	High level	132	(75.00)	102	(80.31)	30	(61.22)	
**METER Test**								
	Low level	71	(40.34)	37	(29.13)	34	(69.39)	<0.001
	Marginal level	84	(47.73)	71	(55.91)	13	(26.53)	
	Adequate level	21	(11.93)	19	(14.96)	2	(4.08)	
**SILS Test**								
	Never	14	(7.95)	13	(10.24)	1	(2.04)	<0.001
	Rarely	52	(29.55)	47	(37.01)	5	(10.20)	
	Sometimes	53	(30.11)	38	(29.92)	15	(30.61)	
	Often	42	(23.86)	21	(16.54)	21	(42.86)	
	Always	15	(8.52)	8	(6.30)	7	(14.29)	

* For categorical variables with multiple categories, *p*-values refer to the overall distribution across categories.

**Table 4:** Univariate and multivariate logistic analysis of the determinants of catch-up vaccination adherence.

Investigated Variables		Univariate Analysis		Multivariate Analysis	
		Crude OR	*p* (z)	Adjusted OR	*p* (z)
**Source of information about vaccines**					
	Primary care pediatrician	3.12	0.001	0.63	0.535
	Media	0.19	<0.001	0.13	0.026
	Health education brochures	4.14	0.025	4.66	0.156
Internet		0.1	<0.001	0.47	<0.001
**Pathologies of the child**					
	Disability	0.05	<0.001	0.01	0.001
	Autism	0.31	0.001	0.57	0.134
**Educational Level**					
	High level	3.43	<0.001	4.76	0.089
**Smoker**					
No, quit smoking more than 10 years ago		0.58	0.399	0.8	0.861
No, quit smoking less than 10 years ago		0.35	0.048	0.09	0.022
	Yes, currently smoking	0.37	0.027	0.69	0.687
**Risk Perception**					
	High level	6.02	<0.001	7.91	0.009
**Positive Outcome**					
	High level	3.8	<0.001	8.62	0.003
**Negative Outcome**					
	High level	6.44	<0.001	1.14	0.849
**Self-efficacy**					
	High level	2.58	0.01	1.78	0.442
**Test METER**					
	Marginal level	5.02	<0.001	4.25	0.113
	Adequate level	7.82	0.005	1.19	0.908
**Test SILS**					
	Rarely	0.72	0.776	0.31	0.112
	Sometimes	0.19	0.131	0.017	0.096
	Often	0.07	0.018	0.017	0.103
	Always	0.08	0.036	0.038	0.226

## Data Availability

Data will be available following a reasonable request to the corresponding author.
